# Prognostic Nomograms Predicting Risk of Keratoconus in Very Asymmetric Ectasia: Combined Corneal Tomographic and Biomechanical Assessments

**DOI:** 10.3389/fbioe.2022.839545

**Published:** 2022-02-17

**Authors:** Xiaoyu Zhang, Lan Ding, Ling Sun, Yangyi Huang, Tian Han, Yishan Qian, Xingtao Zhou

**Affiliations:** ^1^ Eye Institute and Department of Ophthalmology, Eye & ENT Hospital, Fudan University, Shanghai, China; ^2^ NHC Key Laboratory of Myopia (Fudan University), Shanghai, China; ^3^ Key Laboratory of Myopia, Chinese Academy of Medical Sciences, Shanghai, China; ^4^ Shanghai Research Center of Ophthalmology and Optometry, Shanghai, China

**Keywords:** asymmetric ectasia, keratoconus, prognostic nomogram, diagnosis, cornea, biomechanical

## Abstract

**Purpose:** The aim of the study was to develop and validate a prognostic nomogram for subclinical keratoconus diagnosis using corneal tomographic and biomechanical integration assessments.

**Design:** This is a retrospective case–control study.

**Methods:** *Setting:* The study was carried out in a hospital setting. *Patients:* The study included patients with very asymmetric ectasia (VAE) and normal controls. Patients with VAE had defined clinical ectasia in one eye and normal topography (VAE-NT) in the fellow eye, and VAE-NT eyes were selected for analysis. VAE-NT was defined as stratified stage 0 using the ABCD keratoconus grading system. The normal control group was selected from corneal refractive surgery candidates at our clinic, and the right eye was enrolled. *Observation Procedures:* Scheimpflug-based corneal tomography (Pentacam) and corneal biomechanical assessment (Corvis ST) were performed. *Main Outcome Measures:* We performed multiple logistic regression analysis and constructed a simple nomogram via the stepwise method. The receiver operating characteristic (ROC) curve and discrimination and calibration of prognostic nomogram were performed by 500 bootstrap resamplings to assess the determination and clinical value, respectively.

**Results:** A total of 59 VAE-NT and 142 normal eyes were enrolled. For differentiating normal and VAE-NT eyes, the values of specificity, sensitivity, and area under the ROC (AUROC) were 0.725, 0.610, and 0.713 for tomographic parameters, 0.886, 0.632, and 0.811 for biomechanical parameters, and 0.871, 0.754, and 0.849 for combined parameters, respectively. Combined parameters showed better predictability than separated tomographic or biomechanical parameters.

**Conclusion:** Our nomogram developed with combined tomographic and biomechanical parameters demonstrated a plausible, capable, and widely implementable tool to predict risk of keratoconus. The identification of at-risk patients can provide advanced strategies to epitomize ectasia susceptibility.

## Introduction

Early detection of keratoconus is mandatory in candidates for corneal refractive surgery in order to avoid postoperative ectasia and for increased safety in corneal refractive surgery (Binder et al., 2005; Ambrosio and Randleman 2013). The gold standards for screening keratoconus are topographic and tomographic analyses, which are used to detect alterations in corneal morphology, such as thinning, increased curvature, or elevated corneal elevation (de Sanctis et al., 2008; Mihaltz et al., 2009).

Previous studies, which sparked interest in corneal biomechanical assessment, found that biomechanical changes may occur even before tomographic changes (Kozobolis et al., 2012; Tian et al., 2014; Vinciguerra et al., 2016) may appear, and that early diagnosis of biomechanical disorders may provide a new way of detecting *forme fruste* keratoconus and other ectatic corneal diseases. Recently, substantial progress made in the field of *in vivo* corneal biomechanical characteristics has enabled a more robust and accurate biomechanical *in vivo* keratoconus screening and better compensation of the parameters. Corvis ST (OCULUS Optikgeräte GmbH, Wetzlar, Germany) monitors corneal dynamic deformation due to a constant-pressure air pulse using an ultrahigh-speed Scheimpflug camera, and the latest improvement has been the combination of tomographic and biomechanical data derived from Scheimpflug analyses (Pentacam and Corvis ST; OCULUS Optikgeräte GmbH) (Nemeth et al., 2013; Ali et al., 2014). The tomography biomechanical index (TBI) calculated by Pentacam and Corvis ST parameters has provided the possibility of discriminating the tomographical normal eyes from subclinical keratoconus eyes (Vinciguerra et al., 2016; Ambrósio et al., 2017; Ferreira-Mendes et al., 2019).

The purpose of this study was to determine the diagnostic ability of tomographic and biomechanical parameters for keratoconus. We included patients with subclinical keratoconus who demonstrated neither clinical nor tomographic signs of ectasia in one eye and were diagnosed with very asymmetric ectasia (VAE) in the contralateral eye. We developed and validated a prognostic nomogram for diagnosing subclinical keratoconus using corneal tomographic and biomechanical integration assessments.

## Patients and Methods

This retrospective comparative case–control study was approved by the Ethics Committee of the Eye and ENT Hospital of Fudan University (approval number 2021118-1) and adhered to the tenets of the Declaration of Helsinki. All participants were assessed to fulfill informed consent requirements.

### Study Patients

The patients diagnosed with VAE at the Eye and ENT Hospital of Fudan University were enrolled in the study. These patients had defined clinical ectasia in one eye and normal tomography (VAE-NT) in the contralateral eye. Objective tomography for confirming VAE-NT cases included comprehensive ophthalmic examination, normal cornea on slit-lamp examination, the best corrected distance acuity of 20/20 or better, no risk of keratoconus by Pentacam topometric/keratoconus (KC) staging, and stage A0B0C0 by the ABCD keratoconus grading system (Belin and Duncan 2016). The patients with a history of corneal surgery, pregnancy, ophthalmic disease, eye rubbing, or systemic diseases with ocular manifestations were excluded.

A control group with no ocular disorders except refractive errors and no ectasia throughout the 2-year follow-up period was selected from the corneal refractive surgery candidates at our clinic. The right eyes from normal controls were included in this study.

### Corneal Imaging Measurement and Parameters

Scheimpflug-based corneal tomography and corneal biomechanical assessment were performed using Pentacam and Corvis ST (OCULUS Optikgeräte GmbH), respectively. The principles and procedures of these devices have been described in previous articles (Ambrósio et al., 2017; Chan et al., 2018). Both measurements were performed by experienced examiners under the same lighting conditions. The parameters from the Pentacam and Corvis ST were obtained for each eye.

### Statistical Analyses

The characteristics of all included VAE-NT and normal eyes are presented as means (with standard deviations) for continuous variables. One-way analysis of variance and Kruskal–Wallis tests were used to show differences between groups. Multivariate linear regression analysis was used to test the association between the VAE-NT and normal groups.

The receiver operating characteristic (ROC) curves were plotted, and their areas under the ROC curve were calculated. Sensitivity, specificity, positive predictive value, negative predictive value (NPV), diagnostic odds ratio, positive likelihood ratio, and negative likelihood ratio of the applied models were also calculated. The selected tomographic and biomechanical variables were used to construct the predictive models, including multiple fractional polynomial model (MFP model, which allows software to determine whether an explanatory variable was important for the model as well as its functional form), full model, and stepwise selected model (a method of fitting regression models, in which the choice of predictive variables is carried out by an automatic procedure). We also adopted bootstrapping for internal validation (by using 500 bootstrap (BS) resamplings) to verify the models as the relatively small sample size of our study. In the model-development phase, according to the Akaike information criterion, we performed a backward step-down selection process using a threshold of *p* < 0.05, to establish a parsimonious model (stepwise model), and formulated a nomogram.

The model was validated for discrimination and calibration abilities by calculating the probability of each patient in the entire dataset according to the model and comparing it with the actual risk of developing keratoconus. Discrimination was defined as the ability of a model to correctly distinguish between nonevents and events.

The statistical analyses were 2-tailed, and a *p* value < 0.05 was considered statistically significant. All statistical analyses were performed using the statistical packages R (http://www.R-project.org; R Foundation for Statistical Computing, Vienna, Austria) and EmpowerStats (www.empowerstats.com, X&Y Solutions, Inc., Boston, MA, United States).

## Results

### Patient Characteristics and Logistic Regression Analysis of Risk Factors of Keratoconus

A total of 59 eyes with VAE-NT and 142 normal eyes were enrolled in this study. The mean ages were 21.22 ± 6.14 and 27.75 ± 6.96 in the VAE-NT and normal eyes groups, respectively (*p* < 0.001). The percentage of male participants was 79.66% and 35.92% in the VAE-NT and normal eyes groups, respectively.

The baseline corneal tomographic and biomechanical characteristics of the patients are summarized in Table 1. Univariate and multivariate linear regression analyses were performed to determine the association between VAE-NT and normal groups. A total of 27 corneal tomographic variables and 16 biomechanical variables were analyzed to detect subclinical corneal ectasia.

**TABLE 1:** Corneal tomographic and biomechanical parameters and clinical characteristics for normal and VAE-NT eyes.

	Normal	VAE-NT	*p*-value	Univariate		Multivariate	
				OR (95%CI)	*p*-value	OR (95%CI)	*p*-value
Number	142	59		—	—	—	—
Female (N)	91 (64.08%)	12 (20.34%)		—	—	—	—
Male (N)	51 (35.92%)	47 (79.66%)		—	—	—	—
Age (Y)	27.75 ± 6.96	21.22 ± 6.14	<0.001	—	—	—	—
Corneal tomographic parameters							
Kflat (D)	42.64 ± 1.41	42.30 ± 1.40	0.179	0.84 (0.68, 1.05)	0.125	1.12 (0.85, 1.48)	0.422
Ksteep (D)	43.83 ± 1.54	43.62 ± 1.57	0.543	0.91 (0.75, 1.11)	0.373	1.06 (0.83, 1.35)	0.643
Kmax (D)	44.35 ± 1.62	44.27 ± 1.59	0.995	0.97 (0.80, 1.17)	0.735	1.12 (0.89, 1.41)	0.351
BFS anterior	7.91 ± 0.26	7.95 ± 0.27	0.327	2.04 (0.64, 6.50)	0.230	0.66 (0.16, 2.75)	0.569
BFS posterior	6.46 ± 0.24	6.50 ± 0.23	0.269	2.18 (0.60, 7.97)	0.238	0.69 (0.14, 3.34)	0.648
F.Ele. TP (μm)	2.65 ± 1.32	2.73 ± 1.54	0.892	1.04 (0.84, 1.30)	0.706	1.12 (0.86, 1.45)	0.417
B.Ele. TP (μm)	5.44 ± 3.09	6.92 ± 4.37	0.019	1.12 (1.03, 1.22)	0.010	1.11 (1.00, 1.23)	0.055
PPI-min	0.71 ± 0.12	0.77 ± 0.14	0.003	43.82 (3.46, 555.22)	0.004	17.71 (0.98, 321.47)	0.052
PPI-max	1.27 ± 0.16	1.43 ± 0.26	<0.001	56.12 (9.54, 330.23)	<0.001	54.03 (7.15, 408.23)	<0.001
PPI-avg	1.01 ± 0.11	1.08 ± 0.14	<0.001	101.12 (7.64, 1338.44)	0.001	57.03 (3.00, 1083.63)	0.007
ARTmax	429.33 ± 65.88	382.36 ± 76.84	<0.001	0.99 (0.98, 0.99)	<0.001	0.99 (0.98, 1.00)	0.001
Df	0.47 ± 0.96	0.32 ± 1.09	0.264	0.86 (0.63, 1.17)	0.337	1.00 (0.68, 1.45)	0.982
Db	0.08 ± 0.78	0.03 ± 0.92	0.529	0.93 (0.64, 1.35)	0.700	0.96 (0.63, 1.46)	0.845
Dp	0.71 ± 0.76	1.19 ± 0.97	<0.001	1.98 (1.35, 2.91)	0.001	1.83 (1.18, 2.83)	0.007
Dt	0.14 ± 0.75	0.35 ± 0.66	0.056	1.49 (0.96, 2.30)	0.072	1.33 (0.81, 2.17)	0.263
Da	0.54 ± 0.60	0.97 ± 0.70	<0.001	3.23 (1.81, 5.76)	<0.001	2.94 (1.54, 5.59)	0.001
BAD-D	1.11 ± 0.54	1.38 ± 0.72	0.011	2.11 (1.25, 3.58)	0.005	2.32 (1.22, 4.39)	0.010
ARC (3 mm zone) (mm)	7.80 ± 0.26	7.84 ± 0.26	0.431	1.86 (0.58, 5.97)	0.295	0.57 (0.13, 2.47)	0.457
PRC (3 mm zone) (mm)	6.38 ± 0.25	6.33 ± 0.26	0.119	0.45 (0.13, 1.58)	0.213	0.15 (0.03, 0.72)	0.018
Thinnest pachymetry (μm)	533.99 ± 25.90	526.95 ± 22.29	0.058	0.99 (0.98, 1.00)	0.071	0.99 (0.98, 1.01)	0.250
ISV (8 mm Zone)	17.23 ± 4.79	18.05 ± 4.80	0.314	1.04 (0.97, 1.10)	0.267	1.00 (0.93, 1.08)	0.935
IVA (8 mm Zone)	0.12 ± 0.05	0.12 ± 0.06	0.826	2.51 (0.01, 736.22)	0.751	9.11 (0.01, 7263.70)	0.517
KI (8 mm Zone)	1.03 ± 0.02	1.03 ± 0.02	0.342	0.00 (0.00, 6073.86)	0.451	0.81 (0.00, inf.)	0.981
CKI (8 mm Zone)	1.01 ± 0.01	1.01 ± 0.01	0.474	0.00 (0.00, inf.)	0.636	118.12 (0.00, inf.)	0.874
IHA (8 mm Zone)	5.67 ± 4.60	5.42 ± 4.14	0.920	0.99 (0.92, 1.06)	0.721	0.98 (0.91, 1.07)	0.683
IHD (8 mm Zone)	0.01 ± 0.01	0.01 ± 0.01	0.449	11.11 (0.00, inf.)	0.929	1867514.45 (0.00, inf.)	0.640
RMin (8 mm Zone)	7.62 ± 0.28	7.62 ± 0.31	0.909	0.99 (0.34, 2.87)	0.988	0.52 (0.15, 1.89)	0.322
Corneal biomechanical parameters							
A1L	2.30 ± 0.33	2.21 ± 0.36	0.069	0.46 (0.18, 1.15)	0.098	0.49 (0.17, 1.39)	0.180
A2V	0.14 ± 0.02	0.14 ± 0.02	0.868	0.09 (0.00, 98016.29)	0.732	195.81 (0.00, inf.)	0.538
A2L	1.93 ± 0.30	1.83 ± 0.31	0.124	0.33 (0.11, 0.96)	0.042	0.37 (0.11, 1.30)	0.122
A2V	-0.26 ± 0.03	-0.26 ± 0.03	0.671	0.27 (0.00, 10671.37)	0.809	0.30 (0.00, 43403.07)	0.841
Peak distance	4.85 ± 0.27	4.95 ± 0.26	0.024	4.26 (1.26, 14.44)	0.020	4.13 (1.00, 17.09)	0.050
Radius	7.14 ± 0.84	6.79 ± 0.84	0.016	0.60 (0.41, 0.88)	0.009	0.76 (0.48, 1.18)	0.219
Deformation amplitude	1.00 ± 0.08	1.03 ± 0.09	0.022	81.98 (1.83, 3677.67)	0.023	147.64 (1.91, 11421.91)	0.024
IOPnct	17.17 ± 2.85	15.95 ± 2.97	<0.001	0.84 (0.74, 0.96)	0.009	0.86 (0.75, 0.99)	0.042
bIOP	17.33 ± 2.55	16.33 ± 2.76	0.002	0.85 (0.74, 0.97)	0.016	0.84 (0.71, 0.98)	0.024
SSI	0.96 ± 0.15	0.91 ± 0.16	0.014	0.09 (0.01, 0.75)	0.026	0.15 (0.01, 1.62)	0.119
DA ratio (2 mm)	4.30 ± 0.38	4.53 ± 0.51	<0.001	3.55 (1.66, 7.58)	0.001	4.28 (1.75, 10.47)	0.002
Integr. radius	8.38 ± 0.97	8.99 ± 1.09	<0.001	1.87 (1.34, 2.62)	<0.001	1.78 (1.21, 2.61)	0.003
ARTh	469.74 ± 80.21	490.21 ± 112.88	0.316	1.00 (1.00, 1.01)	0.150	1.00 (1.00, 1.01)	0.291
SP-A1	104.68 ± 16.80	107.54 ± 19.32	0.709	1.01 (0.99, 1.03)	0.296	1.01 (0.99, 1.03)	0.513
CBI	0.12 ± 0.20	0.17 ± 0.25	0.688	2.67 (0.69, 10.31)	0.154	2.02 (0.41, 10.02)	0.390
TBI	0.24 ± 0.18	0.40 ± 0.31	0.001	17.67 (4.43, 70.44)	<0.001	14.27 (2.98, 68.34)	0.001

K, keratometry; D, diopters; BFS, best-fit sphere; F.Ele. Th, front elevation at thinnest pachymetry; B.Ele. Th, back elevation at thinnest pachymetry; PPI, pachymetric progression index; min, minimum; avg, average; ARTmax, maximum Ambrósio’s relational thickness; Df, deviation of front elevation difference map; Db, derivation of back elevation difference map; Dp, deviation of average pachymetric progression; Dt, deviation of minimum thickness; Da, deviation of ARTmax; BAD-D, Belin/Ambrósio enhanced ectasia deviation; ARC, anterior radius of curvature; PRC, posterior radius of curvature; ISV, index of surface variance; IVA, index of vertical asymmetry; KI, keratoconus index; CKI, center keratoconus index; IHA, index of height asymmetry; IHD, index of height decentration; RMin, minimum axial/sagittal curvature. A1 L, applanation length at first applanation; A1V, corneal apex velocity at first applanation; A2L, applanation length at second applanation; A2V, corneal apex velocity at second applanation; bIOP, biomechanically corrected IOP; SSI, stress–strain index; ARTh, Ambrósio’s relational thickness in the horizontal profile; DA, deformation amplitude; Integr. radius, integrated radius; SP-A1, stiffness parameter at first applanation; CBI, Corvis biomechanical index; TBI, tomography and biomechanical index.

In the univariate analysis, eight tomographic variables involved with back elevation in the thinnest location, minimum pachymetric progression index (PPI-min), maximum PPI (PPI-max), average PPI (PPI-avg), maximum Ambrósio’s relational thickness (ARTmax), deviation of average pachymetric progression (Dp), deviation of ARTmax (Da), Belin/Ambrósio enhanced ectasia deviation (BAD-D) values, and 10 biomechanical variables involving applanation length at second applanation, peak distance, radius, and deformation amplitude (Def. Amp.), intraocular pressure (IOP) measurements using an automated noncontact tonometer (NCT), biomechanically corrected IOP (bIOP), stress–strain index, deflection amplitude ratio (DA Ratio), and TBI showed significant differences between the two groups. In the multivariate logistic analysis, after adjusting for age and sex, based on the odds ratios (95% CI) and *p* values, PPI-max, PPI-avg, ARTm, Dp, Da, BAD-D, posterior radius of curvature (PRC), peak distance, Def. Amp., IOP using NCT, bIOP, DA ratio, integrated radius, and TBI were significantly different between the two groups.

### Discrimination of the Prognostic Nomogram

**FIGURE 1:**
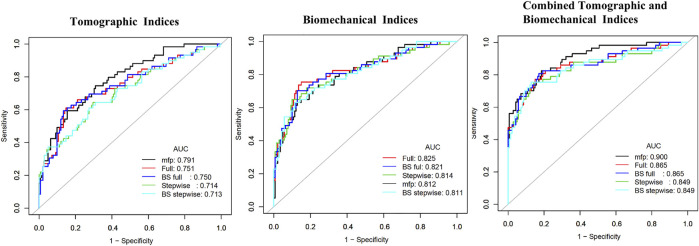
Receiving operating characteristic curve and area under the curve (AUC) in tomographic indices, biomechanical indices, and combined tomographic and biomechanical indices.

The prediction accuracy of tomographic and biomechanical parameters, including ROC curve analysis and optimal threshold analysis, is presented in Table 2, and the ROC curves are shown in Figure 1. The area under the receiver operating characteristic (AUROC) values for the corneal tomographic and biomechanical parameters were 0.713 and 0.811, respectively, for the BS stepwise model. The AUROC for combined tomographic and biomechanical parameters was higher than that of separated tomographic or biomechanical parameters and showed better predictability, reaching 0.900, 0.865, 0.849, 0.865, and 0.849 for the applied MFP model, full model, stepwise model, BS full model, and BS stepwise model, respectively. The optimal cutoff values of the nomogram for the combined tomographic and biomechanical parameters were 0.257, 0.361, 0.337, -1.088, and -0.821 for the applied models, and the specificity rates were 0.814, 0.850, 0.857, 0.829, and 0.871, respectively. The sensitivity percentages were 0.825, 0.772, 0.754, 0.807, and 0.754, respectively (Table 2).

**TABLE 2:** ROC curve analysis and optimal threshold analysis for the predictive model between normal and VAE-NT groups for tomographic parameters, biomechanical parameters, and combined tomographic and biomechanical parameters.

	AUROC	95% CI	Cutoff value	Specificity	Sensitivity	PLR	NLR	DOR	PPV	NPV
Tomographic parameters										
MFP	0.791	0.724 to 0.858	0.396	0.845	0.593	3.829	0.481	7.955	0.614	0.833
Full	0.751	0.672 to 0.829	0.387	0.852	0.610	4.126	0.458	9.019	0.632	0.840
Stepwise	0.714	0.632 to 0.796	0.291	0.704	0.644	2.178	0.505	4.308	0.475	0.826
BS full	0.750	0.6713 to 0.829	-0.454	0.866	0.593	4.434	0.470	9.441	0.648	0.837
BS stepwise	0.713	0.631 to 0.795	-0.855	0.725	0.610	2.222	0.537	4.134	0.480	0.818
Biomechanical parameters										
MFP	0.812	0.745 to 0.880	0.366	0.871	0.632	4.912	0.423	11.619	0.667	0.853
Full	0.825	0.754 to 0.895	0.351	0.850	0.754	5.029	0.289	17.405	0.672	0.895
Stepwise	0.814	0.742 to 0.885	0.403	0.900	0.649	6.491	0.390	16.650	0.726	0.863
BS full	0.821	0.752 to 0.891	−0.642	0.871	0.702	5.458	0.342	15.948	0.690	0.878
BS stepwise	0.811	0.741 to 0.881	−0.302	0.886	0.632	5.526	0.416	13.286	0.692	0.855
Combined tomographic and biomechanical parameters										
MFP	0.900	0.853 to 0.947	0.257	0.814	0.825	4.440	0.216	20.608	0.644	0.919
Full	0.865	0.803 to 0.928	0.361	0.850	0.772	5.146	0.268	19.180	0.677	0.902
Stepwise	0.849	0.782 to 0.917	0.337	0.857	0.754	5.281	0.287	18.429	0.683	0.896
BS full	0.865	0.803 to 0.928	−1.088	0.829	0.807	4.708	0.233	20.212	0.657	0.913
BS stepwise	0.849	0.781 to 0.917	−0.821	0.871	0.754	5.867	0.282	20.818	0.705	0.897

VAE-NT, fellow eye from patients with very asymmetric ectasia with normal tomography; AUROC, area under the receiver operating characteristic curve; PLR, positive likelihood ratio; NLR, negative likelihood ratio; DOR, diagnostic odds ratio; PPV, positive predictive value; NPV, negative predictive value.

**FIGURE 2:**
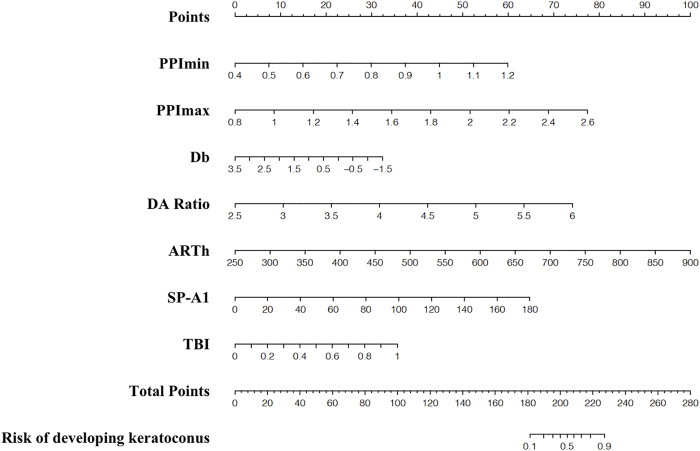
Nomogram for predicting developed-into-keratoconus in VAE-NT patients. VAE-NT, defined clinical ectasia in one eye and normal tomography.

The nomograms for the applied models are shown in Supplementary Material. The nomogram of the stepwise model was drawn to provide a convenient and quantitative tool for predicting the risk of developing keratoconus using PPI-min, PPI-max, derivation of back elevation difference map (Db), DA ratio, Ambrósio’s relational thickness in the horizontal profile, stiffness parameter at first applanation, and TBI, as it was easy to use and generally accepted in clinical practice (Figure 2). To estimate the risk of keratoconus, the values of the different parameters are located on each variable axis. A vertical line is drawn from that value to the top point scale for determining how many points are assigned by that variable value. The points from each variable value are then summed. The sum is located at the total points scale and is vertically projected onto the bottom axis, thus obtaining a personalized risk of keratoconus.

### Calibration of the Prognostic Nomogram

**FIGURE 3:**
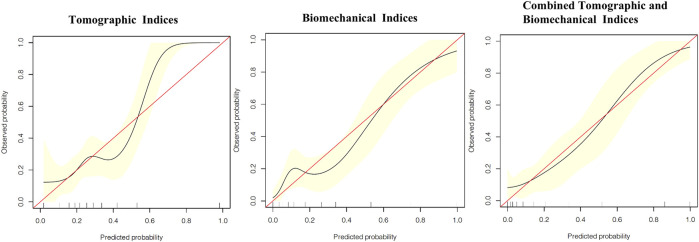
Calibration curves showing the risk of developed-into-keratoconus in VAE-NT cornea according to tomographic indices, biomechanical indices, and combined tomographic and biomechanical indices.

The calibration curves showing the risk of developing keratoconus in the VAE-NT cornea according to tomographic indices, biomechanical indices, and combined tomographic and biomechanical indices are shown in Figure 3, which showed good overall discrimination using the combined tomographic and biomechanical indices nomogram. However, among patients with an actual probability for developing keratoconus of >50%, the model underestimated the risk of keratoconus by ≥ 10%. For an observed keratoconus probability of <50%, the model-predicted probability might be overestimated.

## Discussion

The current study demonstrated the early detection of subclinical keratoconus using Scheimpflug tomography and biomechanical assessments in a specific group of patients diagnosed with VAE to detect valuable diagnostic indexes. We constructed a nomogram-based prognostic model, performed an internal validation, and found that model discrimination effectively evaluated the risk of keratoconus and had a good ability to categorize patients into separate risk strata. To our knowledge, only a few previous studies have applied a prognostic nomogram for the early detection of subclinical keratoconus with combined tomography and biomechanical parameters.

The incidence of keratoconus varies greatly, from 0.5% to 1% (Kennedy et al., 1986; Chan et al., 2021). Increasing morbidity contributes to improved detection methods. With the increasing prevalence of keratoconus, its pathophysiology has been widely discussed. Biomechanical disorders are often considered the main pathogenesis of keratoconus (Ambekar et al., 2011; Wolffsohn et al., 2012; Salomao et al., 2021). Therefore, after ruling out asymmetric external factors causing unilateral keratoconus (Gomes et al., 2015), it is vital to analyze the corneal tomography and biomechanical features of VAE-NT to help clinicians in the early diagnosis of keratoconus and provide a basis for accurate diagnosis.

The shape, ROC, and AUROC were used to estimate the discriminative power of the test. Previous studies have reported good discrimination of AUROC values in individual tomographic and biomechanical parameters. TBI, BAD-D, and CBI have been reported in several published studies that reported the discriminating capability of VAE-NT from normal controls (Ambrósio et al., 2017; Ferreira-Mendes et al., 2019; Kataria et al., 2019; Koh et al., 2019). Some studies reported high AUROC in variables from anterior elevation, including KI, IHD, and IVA (Donoso et al., 2021); however, our study reported other tomographic parameters, including the PPI and Db. This difference can be attributed to the inclusion criteria and racial differences in the anterior segments. Our study enrolled patients who reported no risk of keratoconus by Pentacam topometric/KC staging, which is based primarily on morphological parameters of anterior corneal elevation. A possible confounding factor of asymmetric anterior corneal morphology was excluded and therefore has stronger diagnostic implications for occult keratoconus with normal anterior corneal morphology.

The criterion of “VAE-NT” varies among studies. A comparison of the VAE-NT classification criteria and AUROC of the main outcome measures with previous studies is shown in Table 3. According to the Global Consensus on Keratoconus and Ectatic Disease, posterior corneal elevation is indispensable for the diagnosis of early or subclinical keratoconus (Gomes et al., 2015). Therefore, we referred to the ABCD keratoconus grading system based on the anterior radius of curvature and PRC, thinnest pachymetry, best corrected visual acuity, and a modifier for the presence of corneal scarring and only enrolled VAE-NT patients who were diagnosed with stage A0B0C0D0 (Belin and Duncan 2016). Owing to the restricted inclusion criteria, the ratio of the VAE-NT eyes to normal BAD-D, CBI, TBI, and other parameters might be higher than in other published studies, which led to the diagnostic yield of individual parameter underscores in comparison with a more complex multivariate approach for each parameter. To address this issue, our study combined variables taken from both tomography and biomechanical parameters and demonstrated a high diagnostic capability. The nomogram using combined parameters demonstrated good predictive accuracy, with an AUROC above 0.84 in the applied five models.

**TABLE 3:** Comparison of VAE-NT classify criteria and AUROC of the main outcome measures with previous studies.

Study	VAE-NT classification criteria	Comparison	AUROC of main outcomes	Sensitivity	Specificity
Ambrósio et al. (2017)	Objective front surface curvature metrics derived from Pentacam, such as a keratoconus percentage index (KISA%) score lower than 60 and a paracentral inferior–superior (I-S value) asymmetry value at 6 mm (3 mm radii) less than 1.45	480 normal vs. 94 VAE-NT	TBI: 0.985	TBI: 90.4	TBI: 96.0
			BAD-D: 0.839		
			CBI: 0.822		
Chan et al. (2018)	With an average corneal power of 49.00 D or less or HOAs of 1.50 μm or less in either eye or normal topography but obvious keratoconus in the contralateral eye	37 normal vs. 23 VAE-NT	TBI: 0.925	TBI: 84.4	TBI: 82.4
			BAD-D: 0.786		
Ferreira-Mendes et al. (2019)	Objective front surface curvature metrics derived from Pentacam HR, such as a keratoconus percentage index (KISA%) score lower than 60 and a paracentral inferior–superior (I-S value) asymmetry value at 6 mm (3 mm radii) less than 1.45	312 normal vs. 57 VAE-NT	TBI: 0.960	TBI: 89.5	TBI: 91.0
Kataria et al. (2019)	Objective front surface curvature metrics derived from Pentacam, such as a keratoconus percentage index (KISA%) score lower than 60 and a paracentral inferior–superior (I-S value) asymmetry value at 6 mm (3 mm radii) less than 1.45	100 normal vs. 100 VAE-NT	TBI: 0.901	TBI: 84.0	TBI: 85.0
			BAD-D: 0.812	BAD-D: 87.0	BAD-D: 76.5
			CBI: 0.775	CBI: 68.0	CBI: 72.5
			SPAI: 0.762	SPA1: 66.0	SPA1: 74.5
Steinberg et al. (2018)	KISA% index of less than 60%, I-S difference of less than 1.45 D, and Kmax of 47.00 D or less	105 normal vs. 32 VAE-NT	TBI: 0.825	TBI: 72.0	TBI: 71.0
			BAD-D: 0.748	BAD-D: 69.0	BAD-D: 69.0
			CBI: 0.787	CBI: 69.0	CBI: 69.0
Koh et al. (2019)	Distance-corrected visual acuity of 20/20 or better and a normal Placido-disk topographic map (Klyce/Maeda keratoconus index and Smolek/Klyce keratoconus severity index), based on the definition of *forme fruste* keratoconus	70 normal vs. 23 VAE-NT	TBI: 0.751	TBI: 52.17	TBI: 88.57
			BAD-D: 0.668	BAD-D: 60.87	BAD-D: 85.70
			CBI: 0.660	CBI: 30.43	CBI: 98.57
Koh et al. (2019)	Central mean keratometry value of less than 47.20 diopters (D), an inferior–superior asymmetry for the average keratometry value of less than 1.40 D, a keratoconus percentage index (KISA%) of less than 60%, and no clinical evidence	35 normal vs. 21 VAE-NT	TBI: 0.790	TBI: 67	TBI: 86
Current study	Best corrected distance acuity of 20/20 or better, reporting no risk of keratoconus by Pentacam topometric/KC staging and stage 0 by the ABCD keratoconus grading system	142 normal vs. 59 VAE-NT	BS stepwise model for combined parameters: 0.849	0.754	0.871

Accurate prognostic information for keratoconus is vitally important, not only for determining suitability for corneal refractive surgery, but also for assessing the risk of developing keratoconus in the future. As for the calibration curves, good overall discrimination was achieved using the combined tomographic and biomechanical indices nomogram. For patients with an actual keratoconus probability of greater than 50%, the model underestimated the risk of keratoconus and may result in a reduction of the frequency of follow-up. Our model, with adequate calibration by predicted risk strata, will provide abundant information for clinical decision-making.

It is worth noting that we applied a restricted inclusion criterion, which pooled extensive corneal tomographic and biomechanical data. We also applied discrimination and calibration for the prognostic nomogram, obtaining a robust metric for early diagnosis of keratoconus, which can be applied to a broad range of eyes.

Our study had some limitations, mainly that there were racial differences in the anterior segments, and validation of these tomographic parameters should be noted. Second, combining epithelial thickness mapping with the assistance of anterior segment optical coherence tomography can be useful in future studies. Third, the relatively short postoperative follow-up in the normal control group may not cover the possible development of keratoconus, which may also lead to overestimation of the performance of the ROC analysis. Finally, since the construction and verification of the model were conducted through the single-center database, which may decline the prediction value. A larger sample of clinical data will be needed in the future to externally validate our model in order to improve diagnostic efficacy.

## Conclusion

Several parameters of corneal tomographic and biomechanical examinations can be useful for the early diagnosis of subclinical keratoconus. The prognostic nomogram demonstrated better results when both devices were combined. These results indicate that careful assessment combining different techniques or devices is needed for the detection of subclinical ectasia.

## Data Availability

The raw data supporting the conclusion of this article will be made available by the authors, without undue reservation.
