# Supplementary material for: Prognostic Nomograms Predicting Risk of Keratoconus in Very Asymmetric Ectasia: Combined Corneal Tomographic and Biomechanical Assessments
Source: Front Bioeng Biotechnol. 2022 Feb 17;10:839545. doi: 10.3389/fbioe.2022.839545 (PMC8892177; doi:10.3389/fbioe.2022.839545)
Supplement: Supplementary file 1 [file DataSheet1.DOCX]

**Supplementary materials**

**Supplementary material 1.** Nomogram for applied models

**Nomogram for MFP model**

-24.64779 +14.22781*I((ARTh/1000)^1) +4.13883*I((TBI + 0.1)^2) +8.32148*I(PPImin^1) -0.94834*I((Db + 1.5)^1) +3.63216*I(PPImax^1) +25.21526*I((DA Ratio/10)^1) +1.07907*I(((B.Ele.TP + 1)/10)^1) -0.22901*I((ISV/10)^1) -3.05381*I(((Axis + 0.1)/100)^2) +5.65383*I(((Axis + 0.1)/100)^2 * log(((Axis + 0.1)/100))) -2.96733*I((A1V/0.1)^1)

**Nomogram for Full model**

-8.33877 -0.10380*Kflat +0.00120*Axis +0.11606*B.Ele.TP +6.47592*PPImin +3.08693*PPImax +0.18374*Df -0.84733*Db +0.02015*Dt -0.03588*ISV -6.75262*KI -3.21944*CKI -0.01721*IHA -31.05899*IHD -0.41888*A1L -3.25388*A1V -0.47353*A2L +6.52529*A2V +0.57242*Peak Distance

 -0.21814*Radius -1.62016*SSI +1.50291*DA Ratio +0.01366*ARTh +0.02927*SP-A1 +0.51119*CBI +4.07013*TBI

**Nomogram for Stepwise model**

-32.36317 +7.18074*PPImin +4.12830*PPImax -0.62210*Db +2.03104*DA Ratio +0.01476*ARTh +0.03452*SP-A1 +3.42003*TBI

**Nomogram for BS full model**

-8.53894 -0.17766*Kflat +0.00245*Axis +0.20915*B.Ele.TP +8.73899*PPImin +3.81425*PPImax +0.23814*Df -1.43502*Db -0.01871*Dt -0.05352*ISV -6.14478*KI -15.12571*CKI -0.02330*IHA -46.93185*IHD -0.52755*A1L -0.29056*A1V -0.58577*A2L +9.59407*A2V +1.22654*Peak Distance -0.32417*Radius -1.38806*SSI +2.54055*DA Ratio +0.01700*ARTh +0.05702*SP-A1 +0.67306*CBI +5.94889*TBI

**Nomogram for BS stepwise**

-42.86203 +8.16539*PPImin +4.31647*PPImax -0.98449*Db +3.11247*DA Ratio +0.01600*ARTh +0.07060*SP-A1 +3.90016*TBI

(K, Keratometry; B.Ele. Th, back elevation at thinnest pachymetry; PPI, pachymetric progression index; min, minimum; max, maximum;Df, deviation of front elevation difference map; Db, derivation of back elevation difference map; Dt, deviation of minimum thickness; ISV, index of surface variance; KI, keratoconus index; CKI, center keratoconus index; IHA, index of height asymmetry; IHD, index of height decentration; A1L, applanation length at first applanation; A1V, corneal apex velocity at first applanation; A2L, applanation length at second applanation; A2V, corneal apex velocity at second applanation; ARTh, Ambrósio’s relational thickness in the horizontal profile; DA, dedormation amplitude; SP-A1, stiffness parameter at first applanation; CBI, corvis biomechanical index; TBI, Tomography and Biomechanical Index.)
